# Muscle Oxygenation Unlocks the Secrets of Physiological Responses to Exercise: Time to Exploit it in the Training Monitoring

**DOI:** 10.3389/fspor.2022.864825

**Published:** 2022-03-07

**Authors:** Stephane Perrey

**Affiliations:** EuroMov Digital Health in Motion, Univ Montpellier, IMT Mines Ales, Montpellier, France

**Keywords:** training, NIRS, muscle, monitoring, internal load

## Introduction

Our understanding of the human physiology during exercise lags behind our understanding of human behavior for decades. However, the number of available technologies for monitoring physiological functions “in the wild” and for assessing various training related-parameters has increased dramatically in recent years. In order to maximize exercise performance, mitigate fatigability as well as minimize the risk of injury, coaches and practitioners need a tough understanding of the relationship(s) between the external work outputs and its physiological impacts among other performance determinants (Vallance et al., 2020). Quantifying of how muscles respond to physical exercise conditions with sufficient accuracy and response time is important in athletes to unveil in dynamic fashion the adaptations to the training bouts and potential limiting factors of exercise performance. In exercise physiology, measurements of systemic changes (i.e., heart rate, HR; oxygen consumption, VO2; blood lactate) in the body are widely used in the prescription of exercise intensity and monitoring of physical conditioning. Besides, monitoring of the working muscles was proposed for tracking the muscle physiology adaptability during exercise, but it still remains nascent and not so used in the athletic field (Perrey and Ferrari, 2018). However, there is a strong demand in the monitoring of athlete training to use alternative indicators or metrics, to allow drawing of an accurate and reliable reflection of muscle status over time. Muscular activation (recorded by surface electromyography) and muscle oxygenation (assessed by near infrared spectroscopy, NIRS) are considered as the main approaches in the monitoring of working muscle (Ferrari et al., 2011). While electromyographic technique represents the electrical activity generated in muscle fibers, NIRS aims to assess the local oxygenation responses reflecting the balance between oxygen (O_2_) delivery and utilization at the level of the microvasculature within skeletal muscle. In 2019, Barstow proposed a deep physiological explanation of the NIRS technique, and described its strengths, limitations and a few applications to skeletal muscle research during common exercise modalities (i.e., incremental exercise, square wave transitions). The NIRS technique is based on the light absorption of oxygenated and deoxygenated hemoglobin (Hb) and myoglobin (Mb) in the near infrared tissue, using the interaction of light at different wavelengths. It allows to measure the changes in oxyhemoglobin (O2Hb), deoxyhemoglobin (HHb), total hemoglobin, and tissue saturation index (StO2) in skeletal muscle during exercise. These last years, small wireless and portable NIRS devices have been built for sports applications.

Thus, NIRS technique presents an exciting opportunity to measure human physiological indicators in a continuous, real-time, and non-intrusive manner. Current wearable NIRS monitors (e.g., Moxy, BSI Insight, and Portamon devices) growing in availability, offer promising approaches but formal longitudinal and cohort studies are still needed to assess their use-case for sports (Perrey and Ferrari, 2018). In this context, allowing continuous and sensitive monitoring with high frequency of NIRS signal capture may contribute to the improvement of the organization of exercise and training load monitoring aimed at improving performance.

This opinion article concentrates on the benefits of utilizing NIRS technique in measuring the muscle physiological status and determining how the working muscles are being exerted. Some questions are addressed: are NIRS-derived hemodynamic variables able in delineating different levels of physical workload? Are these measures sufficiently sensitive to varying changes in exercise conditions (e.g., intensity, terrain)? What are the current challenges?

## A Plethora of Possibilities for Prescribing and Controlling Exercise Intensity

Systemic responses, such as VO2 and blood lactate, which are associated with intramuscular changes, are typically used as indicators of homeostatic perturbations in response to different exercise intensities. An alternative approach for prescribing exercise intensity could be based on a local indicator providing specific information about the working muscle(s) status. These last two decades, much attention has been devoted primarily to muscle oxygenation time course analysis during incremental exercise tests (Bhambhani et al., 1997). It has been emphasized that NIRS measurements may represent a simple, safe, reliable, and fast way to determine training intensity zones based on the determination of several thresholds representing shifts in the metabolic status of the working muscle (Grassi et al., 1999; Racinais et al., 2014; Borges and Driller, 2016; Crum et al., 2017; Rodrigo-Carranza et al., 2021). Those studies observed moderate to high correlations between NIRS breakpoints and traditional thresholds detection methods (i.e., the gas exchange, the first and second lactate or ventilatory thresholds). Reproducibility for NIRS derived breakpoints is however still limited to laboratory setup (see Barstow, 2019). In the field, on the one hand, these breakpoints might be used as a valid surrogate of ventilatory and/or lactate thresholds while being assessed on a regularly basis according to the training phases. Accordingly, based on a systemic physiological approach, muscle oxygenation recording should be implemented further by coaches and practitioners as portable devices for monitoring changes in the workload at various threshold levels in endurance training (Driller et al., 2016). Data collection with gas exchange measurement requires tedious and unique laboratory equipment while multiple blood sample withdrawal for lactate threshold is rather inconvenient for the athletes and the staff, especially if larger numbers of athletes are tested. In the daily training environment, reducing the number of devices, and reducing installation time before every test is important. It means that with NIRS the use of only one muscle (primary locomotor muscle) should be target according to the specificity of the task. Obviously, it might be an oversimplification of the underpinning and complex whole-body physiology to exercise. On the other hand, based on a more local physiological approach (e.g., fiber type distribution), expected heterogeneity of response profiles across different muscle sites (upper vs. lower body, locomotor vs. non locomotor, and among locomotor muscles) should be considered according to the physical task demands. Such multiple NIRS breakpoints metrics may allow for a better categorization of training stimulus into zones of exercise intensity for specific muscle groups (resistance/strength training), and in turn will favor the expected local muscle adaptations. In short-endurance event, oxygenation in *biceps brachii, latissimus dorsi* and *vastus lateralis* muscles was shown to be more relevant than VO2max in predicting canoe-kayak performance (Paquette et al., 2018).

Demarcating exercise intensity zones can be assessed with other metrics, such as the critical power/speed derived from the power-duration relationship. For constant-load heavy exercise, the maximal lactate steady state and critical power/speed were determined accurately by measuring the level of deoxygenated hemoglobin or StO2 (Snyder and Parmenter, 2009; Bellotti et al., 2013; Feldmann and Erlacher, 2021). Since critical power, the greatest metabolic rate achieved through the oxidative pathways, is influenced by the balance of oxygen delivery and utilization in working muscles, muscle oxygenation may be used to approximate the power-duration relationship in real-time. Based on a physiological framework, critical oxygenation as a counterpart of critical power method calculated at various workloads and over various durations was proposed by Feldmann and Erlacher (2021). This new individual metric can be useful in the understanding of the origin of muscle performance and fatigue for various sporting disciplines. Interestingly, the balance between muscle O_2_ supply and metabolic demand as a critical metabolic rate in two sites (quadriceps and forearm), allows for predicting time to exhaustion during continuous and intermittent exercise (Kirby et al., 2021).

## A Better Insight of the Internal Training Load

The goal of physical training is to deliver the appropriate physiological stimuli (volume^*^intensity^*^frequency) to achieve adaptations. As a consequence, accurate assessment and monitoring of training load is of paramount importance. To quantify the training load, both external (amount and quality of workload performed) and internal (the individual psycho-physiological responses to the external workload imposed) indicators can be used (Vallance et al., 2020). To date, there is a need for future advancements toward precision training monitoring for dynamic adjustment based on the individual response to physical workouts. However, while perceptual (rating of perceived exertion, RPE) and physiological (systemic variables) metrics are often proposed for evaluating athlete's response to exercise, it is currently not clear which physiological indicator(s) with sufficient sensitivity to exercise loading is/are the most suitable to target. Traditional physiological indicators used are blood lactate and HR measurements. Regarding lactate, its measure requiring capillary blood to be collected at limited epochs is a reliable metabolite occurring during steady-state exercise conditions. Although HR measure has been commonly used, some limitations do exist. Literature has shown relatively low usefulness of HR measurements in strength training, interval endurance training, and when training in naturally hilly terrain (uphill and downhill sections). Furthermore, HR depends on many factors related to emotional state, degree of rest and hydration, and environmental conditions (temperature, humidity, time of day, caffeine, altitude, etc.). One of the main limitations of HR monitoring in athletes appears to be the low sensitivity of HR response to some exercise features. Indeed, while systemic physiological (HR and VO2) responses are characterized by a certain lag (Perrey et al., 2001), the local deoxygenation is responding faster to changing exercise intensity in control lab setup (Bringard and Perrey, 2014). Recent field studies showed that muscle NIRS measurement can give a better indication of exercise intensity than HR across varied terrain (trail running in Born et al., 2017; long-distance cross-country skiing race in Stöggl and Born, 2021). Altogether, these findings suggest that NIRS may provide a robust method to track rapid changes of muscle activation and its metabolic status in exercise conditions (e.g., changes in altitude and locomotion speed), as well as a more detailed information of training potential by discerning upper and lower body muscles participation. Using multiple NIRS sensors to different muscle groups, comparing local metabolic responses among each muscle toward the energy demand of the exercise is possible, and may help to diagnose weakness and strengths within a tailored training approach. Additionally, NIRS devices allow for the measurements in the applied settings as evidenced for a while in various challenging and even extreme sports such as speed skating (Hesford et al., 2013), swimming (Jones et al., 2018), and kayaking (Paquette et al., 2018–2020). In aquatic environments with a specially waterproofed NIRS device, Jones et al. (2018) observed that improved oxidative metabolism of the muscle following 8-weeks endurance training in adolescents was strongly associated (not for HR and RPE) with the improved swim exercise performance. Due to rapid time resolution with NIRS, practical insights of changes in oxygenation and blood volume were observed also during typical swim movements (sprints, turns). In junior kayakers, peripheral adaptations, as assessed *via* NIRS-derived changes in muscle oxygenation (for upper- and lower-body muscles), appear stronger predictors of kayak performance compared to VO2max in both short and long events (Paquette et al., 2018). Noteworthy, benefit of using portable NIRS as a monitoring tool to track 3-weeks training effects on muscle oxygen extraction was even highlighted in male elite senior kayakers (Paquette et al., 2020) although important variability in muscle oxygenation response to training occurs. In summary, it means that the easy implementation of such technology purporting to the specificity of the sports in the field, should be driven by a rethinking of internal load characteristics according to the context. Due to its better sensitivity, a prescription of exercise intensity using NIRS-derived muscle parameters should be implemented in a manner comparable to routinely used conventional internal parameters (e.g., HR, lactate, VO2, RPE). As reported previously, the practicability and effectiveness of this approach has been tested successfully in various acute exercise setting, but is currently underutilized over a competitive season.

## Current Challenges and Perspectives

To date, there are still obstacles plaguing the widespread use of portable NIRS sensors in sports settings. The development of NIRS applications during exercise faces some challenges including methodological considerations and the knowledge of the collected metrics on what constitute a good interpretation. The first main limitation to all NIRS devices is the reduced sensitivity (due to scattering attenuation) to muscles in the presence of subcutaneous adipose tissue where thin adipose layers provide larger changes in StO2 values (Ferrari et al., 2011). Personalized correcting for this attenuation has been suggested based on the strength of the relationship between NIRS-derived measurements and the adipose tissue thickness using muscle site-specific factors (Craig et al., 2017). Second, the hemoglobin concentration spatial distribution throughout the muscle needs to be considered with NIRS (Hamaoka et al., 2011); muscle oxygenation of the most relevant muscle groups for the physical activity can be tracked. Third, it is important to consider the potential increase in skin blood flow associated with prolonged exercise (Ferrari et al., 2011), inducing increased muscle oxygenation values (Davis et al., 2006). All these limitations can be tackled through current and next developments, which will increase the NIRS practical usefulness. Finally, the assessment of muscle status based on NIRS data requires computational tools explaining the observed intra- and inter-individual variability responses to exercise and training programs. With NIRS monitoring, the continuous traces collected could be analyzed *via* time-series segmentation to detect how the working muscle status is evolving as a function of time. Implementation of evidence-based training framework should lead to the development of basic analysis for NIRS indicators (StO2, reoxygenation rate, blood flow) and methods for diagnosing muscle oxygenation in a personalized manner and identifying responders/non-responders to exercise bouts. Modeling the inter-individual differences of muscle oxygenation and the intra-individual responses according to the muscle groups in responses to exercise would lead to propose more accurate exercise prescription. Machine learning and data-driven artificial intelligence approaches can help to achieve this goal. Last potential limitation to consider may be for most practitioners and club the relatively substantial cost of current NIRS devices although their costs have dramatically decreased during recent years, as compared to HR monitors.

## Conclusion

To continuously monitor muscle oxygenation during exercise in the field, small portable NIRS devices have been built. Despite important advances in athlete monitoring technologies, few muscle NIRS devices are currently used in sports settings. But NIRS is able for assessing two of the major determinants of exercise capacity: oxygen delivery and oxygen utilization. The reliability of the microvascular oxygenation measurements observed in the literature, along with the portability of the wireless NIRS device, not too cumbersome (small size and lightweight) for the athlete suggest that it may be very useful for measuring changes in local microvascular oxygenation in diverse sports settings. Portable NIRS devices provide vital insights into how muscles are exerting during training that are not provided by other systemic metrics. In addition, it can be used to compliment other external load metrics (e.g., distance, power, speed, force) to give a clearer picture during training. NIRS devices could provide a collection of athlete internal load data, whereby coaches and practitioners can use various derived oxygenation metrics to monitor, evaluate, and prescribe training (and competition) characteristics. It is now time to integrate muscle oxygenation metrics beside the most common methods (i.e., HR and RPE) for quantifying internal training loads.

## Author Contributions

The author confirms being the sole contributor of this work and has approved it for publication.

## Conflict of Interest

The author declares that the research was conducted in the absence of any commercial or financial relationships that could be construed as a potential conflict of interest.

## Publisher's Note

All claims expressed in this article are solely those of the authors and do not necessarily represent those of their affiliated organizations, or those of the publisher, the editors and the reviewers. Any product that may be evaluated in this article, or claim that may be made by its manufacturer, is not guaranteed or endorsed by the publisher.
